# Physician estimation of the prevalence and clinical impact of chronic urticaria: results of the global, multicenter UCARE CU-PAPER study

**DOI:** 10.3389/falgy.2025.1732893

**Published:** 2026-01-12

**Authors:** Berenike M. Kern, Felix Aulenbacher, Ivan Cherrez-Ojeda, Jason E. Hawkes, Emek Kocatürk, Philip H. Li, Iman Nasr, Pascale Salameh, Hanna Bonnekoh, Pavel Kolkhir

**Affiliations:** 1 Department of Allergy, Institute of Allergology, Charité—Universitätsmedizin Berlin, Corporate Member of Freie Universität Berlin and Humboldt-Universität zu Berlin, Berlin, Germany; 2 Fraunhofer Institute for Translational Medicine and Pharmacology ITMP, Immunology and Allergology, Berlin, Germany; 3 Allergy and Pulmonology Department, Universidad Espiritu Santo, Samborondon, Ecuador; 4 Allergy and Pulmonology Department, Respiralab Research Group, Guayaquil, Ecuador; 5 Oregon Medical Research Center, Portland, OR, United States; 6 Oregon Health & Science University, Portland, OR, United States; 7 Department of Dermatology, Bahçeşehir University School of Medicine, Istanbul, Türkiye; 8 Division of Rheumatology & Clinical Immunology, Department of Medicine, The University of Hong Kong, Hong Kong, Hong Kong SAR, China; 9 Immunology and Allergy Unit Royal Hospital, Muscat, Oman; 10 Faculty of Pharmacy, Lebanese University, Hadat, Lebanon; 11 Department of Research, Gilbert and Rose-Marie Chagoury School of Medicine, Lebanese American University, Beirut, Lebanon; 12 Department of Primary Care and Population Health, University of Nicosia Medical School, Nicosia, Cyprus; 13 Research Methods Department, Institut National de Santé Publique D'Épidémiologie Clinique et de Toxicologie-Liban (INSPECT-LB), Beirut, Lebanon

**Keywords:** chronic inducible urticaria, chronic spontaneous urticaria, disease burden, physician estimation, prevalence

## Abstract

**Introduction:**

Chronic urticaria (CU) is a common, burdensome mast cell-mediated skin disease. However, little is known about physician estimation of the disease prevalence and clinical impact. This study assessed physicians’ perceptions of CU and compared them with data from peer-reviewed literature.

**Methods:**

We conducted a cross-sectional, questionnaire-based study distributed worldwide via UCARE (Urticaria Centers of Reference and Excellence) network to 198 UCAREs and forwarded to GPs (general practitioners). Questionnaire data was analyzed using SPSS and subgroup analysis was performed using appropriate non-parametric statistical tests depending on the number of comparison groups and the distribution of the data.

**Results:**

In total, 234 participants from 46 countries completed the survey [54.3% female, median age: 48.5, interquartile range (IQR) 38.3–58.0 years]. Median age of disease onset reported for chronic spontaneous urticaria (CSU) and chronic inducible urticaria (CIndU) was 30.0 and 25.0 years, respectively. Median estimated prevalence of CU in adult and pediatric patients was 2.5% and 1.0%, respectively. The most affected aspects of patient life, estimated by physicians, were sleep (CSU), physical activities (CIndU), and mental status (CSU, CIndU). Physicians working at UCARE (78.9% (183/232) vs. non-UCARE (21.1% (49/232) sites differed in their estimation of adult CU prevalence (median: 2.0% vs. 4.5%, *p* < 0.001), proportion of adult CSU patients among CU patients (median: 70.0% vs. 60.0%, *p* = 0.027), and proportion of CSU patients with severe impact (median: 50.0% vs. 30.0%, *p* = 0.042). When asked how confident participants were in their estimations regarding CU prevalence and burden, UCARE experts reported significantly higher median confidence compared to other physicians.

**Conclusion:**

Although physicians’ estimations of CU prevalence and burden generally align with literature data, non-UCARE physicians may underestimate the burden and overestimate the prevalence. This might affect CU management in primary care potentially leading to a less effective treatment and underscores the need for increased urticaria awareness among non-UCARE physicians via publications, masterclasses, webinars, or other educational initiatives.

## Introduction

Chronic urticaria (CU) is an inflammatory, mast cell-mediated skin disease characterized by wheals, angioedema, or both for more than 6 weeks. CU occurs spontaneously (chronic spontaneous urticaria, CSU) and/or is triggered by specific external stimuli such as cold and skin scratching (chronic inducible urticaria, CIndU) (1–3). Most studies reported the point prevalence of CU in adults and children <2% (4), with middle-aged females more frequently affected (5). The point prevalence of CSU, CIndU, or CSU and CIndU in the same patient (CSU + CIndU) in the general population is ∼0.02%–2.7%, 0.05%–1.5%, and 0.05%, respectively, corresponding to approximately 80 million affected individuals worldwide (2, 4, 6–8). The age of onset of CSU is approximately 30–50 years, whereas the median age of onset of CIndU is 20–35 years (3).

CU is debilitating and challenging to manage. Second generation H1-antihistamines at approved or increased doses (up to four-fold standard dosing) lead to complete disease control (Urticaria Control Test, UCT = 16) in less than 10% of CU patients (9). The second-line treatment with omalizumab, an anti-IgE monoclonal antibody, leads to complete disease control in about 30% of patients with antihistamine-refractory CSU (9, 10). High rates of sleep impairment, psychiatric, and other comorbidities in CU patients further contribute to the overall disease burden, considerable impairment of patient's quality of life, and high healthcare costs (11, 12).

Physicians’ perceptions of the disease can influence disease management, impacting treatment decisions, adherence, and overall patient outcomes. For example, underestimation of disease severity and burden can lead to biased or suboptimal care, potentially affecting patient well-being and quality of life (13). However, CU prevalence and burden studies were based primarily on epidemiological studies and data provided by patients. Little is known about how physicians perceive CU or assess CU prevalence and disease impact (3, 14, 15). CU-PAPER (Chronic Urticaria—Physician Estimation of Prevalence and Clinical Impact) is a multicenter, cross-sectional study of Urticaria Centers of Reference and Excellence (UCARE) that aims to address these gaps by assessing physicians’ estimations of the prevalence and clinical impact of CU and comparing them to published data. We also investigated whether different groups of physicians have a different view on CU disease prevalence and burden.

## Methods

### Data collection

This cross-sectional study used online questionnaire in “REDCap” (Nashville, Tennessee), a secure, web-based software platform designed to support data capture for research studies (https://www.project-redcap.org) (16, 17). The link to the survey was sent to 198 UCARE centers through a UCARE email distribution list. UCARE experts were also asked to forward the questionnaire to GPs who manage patients with urticaria in their respective countries. This project was also publicly presented at the Global Urticaria Forum (GUF) in December 2024 in Berlin, Germany. Attendees were invited to participate via a QR code displayed on a presentation slide. A reminder email was also sent to 365 individual email addresses contained in a UCARE network contact list to increase survey response rates. Responses were collected between September 5, 2024, and February 25, 2025.

### Ethics approval

CU-PAPER was approved in 2024 by the Ethics Committee of the Charité — Universitätsmedizin Berlin, Germany (reference EA2/130/24).

### Questionnaire

The questionnaire included five different sections (Supplementary Figure S1). The first section asked about professional and demographic information. Section two inquired whether the respondents managed patients with CU in their clinical practice and whether they managed adult patients (>18 years), pediatric patients, or both. The third section included 10 questions about CU: (I) Estimation of the average age of disease onset, asked separately for CSU, CIndU, and CSU + CIndU; (II) level of confidence in their estimations about disease onset; (III) estimation of the prevalence (%) of CU in adults in their country; (IV) estimated proportions of CSU/CIndU among CU patients; and (V) confidence in these prevalence and proportions estimations. The same questions were then asked for pediatric patients. In section four, respondents were asked about their perception of the aspects of a patient's life that were most affected by CSU, CIndU, and CSU + CIndU on a scale from 1 (most affected) to 8 (least affected), what the proportions of low/moderate/high impact of CSU, CIndU, and CSU + CIndU on their patient's lives were, and to indicate how confident they were in their estimations. For respondents who reported having CU, a fifth section asked questions on physicians’ own symptoms including type of CU, disease activity, and most frequent and bothersome symptoms.

### Statistical analysis

Minimal sample size calculation was performed using the G*Power software (18). Based on the primary objective, which was to compare physicians’ perception of urticaria prevalence/incidence to the ones reported in the literature, and considering a small to moderate effect size g of the difference between the perceived and the true measure (g = 0.1), an alpha of 5% and a beta of 20%, the minimal sample size would be *n* = 199. Moreover, based on secondary objectives of comparing subgroups of physicians and finding the correlates of urticaria perception, as the major dependent variables are quantitative, multiple regressions would be used to assess their correlates. Assuming a calculated effect size is f2 = 0.11 (small to moderate effect size) and expecting a squared multiple correlation of 0.1 (R2 deviation from 0) related to the Omnibus test of the multiple regression, the minimum necessary sample is *n* = 205, considering an alpha error of 5%, a power of 80%, and allowing 20 predictors to be included in the model. Thus, a minimal sample size of 225 physicians was targeted, taking into account possible missing values. Continuous variables were reported as median (IQR) and categorical variables as frequency (%). To examine the estimated impact of CSU, CIndU, and CSU + CIndU on daily life, a one-sample t-test was conducted using the mean of the most impaired domain as a reference value. Higher scores, therefore, indicate that an area was estimated as less affected than the most affected domain. To assess differences in continuous or ordinal variables between independent groups, appropriate non-parametric statistical tests were applied depending on the number of comparison groups and the distribution of the data. The normality of continuous variables was evaluated using the Shapiro–Wilk and Kolmogorov–Smirnov tests. The Mann–Whitney *U*-Test was used to compare differences between two independent groups when the dependent variable was either ordinal or continuous but did not meet the assumption of normality. The Kruskal–Wallis H Test was employed when comparing more than two independent groups on an ordinal or continuous outcome variable that violated the assumptions of normality and homogeneity of variance. All statistical analyses were conducted using SPSS Version 30.0.0.0 (build 171). A two-sided *p*-value of less than 0.05 was considered statistically significant. The Figures were created using GraphPad Prism 9 [Version 9.5.0 (525), GraphPad Software, San Diego, California, USA].

## Results

### Demographic data

In total, 234 participants from 46 countries completed the survey (54.3% female, median age: 48.5, IQR 38.3–58.0 years) (Table 1, Supplementary Figure S2, Supplementary Table S1). Most participants were from Europe (44.9%, 105/234), Asia (37.2%, 87/234), and South America (12.4%, 29/234). Most of them were allergist/clinical immunologists (54.7%, 128/234) or dermatologists (38.9%, 91/234) with a median clinical experience of 20.0 years (IQR 10.0-30.0). Approximately two-thirds (68.1%, 158/232) of participants worked in a university/training hospital with 78.9% of these institutions being a UCARE center. Most participants reported seeing children and adult patients with CU (69.0%, 160/232), whereas 24.1% (56/232) and 4.3% (10/232) reported seeing only adults and children, respectively (Table 1). Of the 226 surveyed physicians, 52.2% (118/226) and 15.5% (35/226) stated that they had been affected by acute urticaria and CU, respectively. Among the 35 physicians affected by CU, 40.0% (14/35) had CSU, 37.1% (13/35) CIndU, and 22.9% (8/35) had both. The median CU duration in affected participants was 5.0 years (IQR 2.0-10.0) (Supplementary Table S3).

**Table 1:** Demographic data of participating physicians.

Parameter		Results
Region, % (*n*)	Number of physicians who indicated a region of residence	234
	Africa	1.7 (4)
	Asia	37.2 (87)
	Europe	44.9 (105)
	North America	3.8 (9)
	Oceania	0.0 (0)
	South America	12.4 (29)
Gender, % (*n*/*N*) female		54.3 (127/234)
Age in years, median [(IQR), *N*]		48.5 (38.3–58.0), 220
Age groups in years, % (*n*)	25–35	16.8 (37)
	36–50	37.7 (83)
	51–65	39.6 (87)
	66 or older	5.9 (13)
Specialty, % (*n*)	Total number of replies	234
	Allergy/allergology or allergy/clinical immunology	54.7 (128)
	Dermatology	38.9 (91)
	General practice/primary care	4.3 (10)
	Internal medicine	1.3 (3)
	Pediatrics	0.9 (2)
Years in clinical practice, median [(IQR), *N*]		20.0 (10.0–30.0), 232
Primary place of work, % (*n*)	Total number of replies	232
	University/training hospital	68.1 (158)
	Public hospital	9.1 (21)
	Private hospital	7.3 (17)
	Private practice (office)	13.4 (31)
	Other (family physician/private clinic/outpatient clinic/ pharmaceutical Industry/residency program)	2.1 (5)
Do you see patients with CU, % (*n*)	Total number of replies	232
	No	2.6 (6)
	Only adult patients	24.1 (56)
	Only pediatric patients	4.3 (10)
	Both adult and pediatric patients	69.0 (160)
UCARE % (*n*/*N*) yes		78.9 (183/232)
AU personally affected % (*n*/*N*)		52.2 (118/226)
CU personally affected % (*n*/*N*)		15.5 (35/226)

IQR, interquartile range; *N*, total group size; *n*, subgroup within the total group; AU, acute urticaria; CU, chronic urticaria; UCARE, urticaria centers of reference and excellence.

### Estimation of CU onset and prevalence

Median estimated age of disease onset reported for CSU, CIndU, and CSU + CIndU was 30.0 years (IQR 25.0-40.0), 25.0 years (IQR 20.0-30.0) and 30.0 years (IQR 25.0-35.0), respectively (median confidence: 60.0%, IQR 40.0-75.0). The median estimated prevalence of CU in adult patients was 2.5% (IQR 1.0-5.0). The median estimated proportion of CSU patients among all adult CU patients was 70.0% (IQR 55.0-80.0), the proportion of concomitant CIndU in adults with CSU was estimated to be 30.0% (IQR 20.0-45.0; median confidence: 64.0%, IQR 50.0-75.0). The median estimated prevalence of CU in children was 1.0% (IQR 1.0-3.0). The median estimated proportion of CSU patients among all children affected by CU was 60.0% (IQR 29.8-78.5), and the estimated proportion of concomitant CIndU in children with CSU was 29.5% (IQR 11.0-50.0; median confidence: 57.5%, IQR 32.0-71.0) (Supplementary Table S2).

### Estimation of CU burden

**Figure 1:**
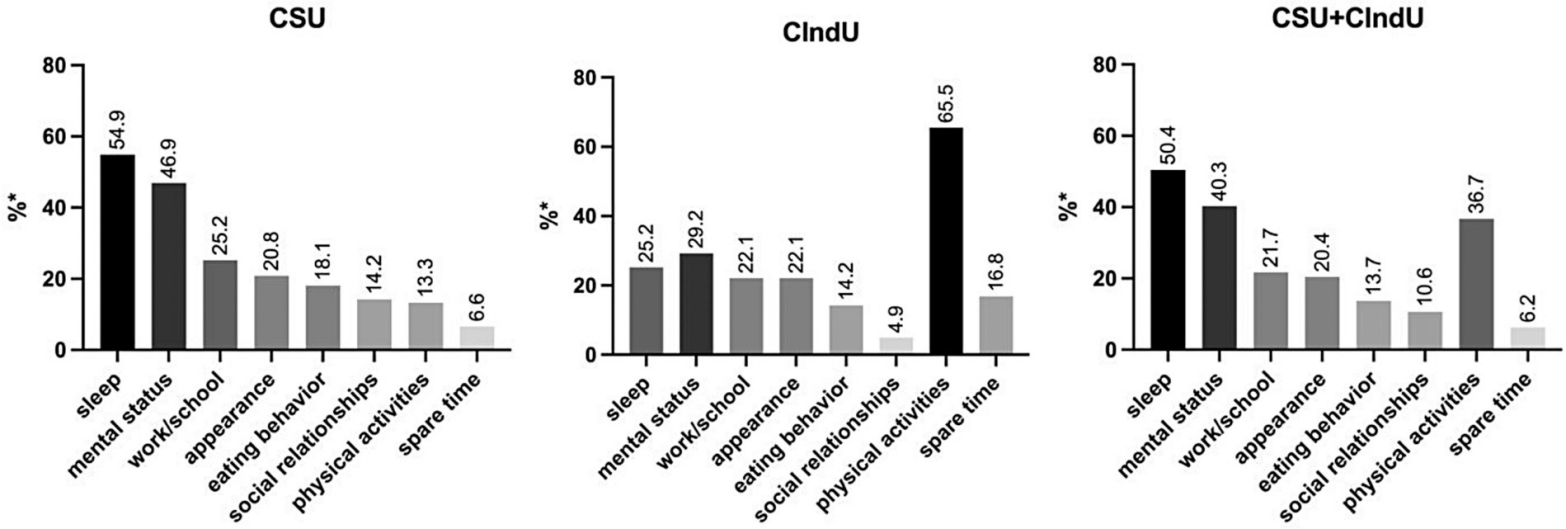
Estimation of CU burden. * % of physicians who rated the area as most or second most affected. CU, chronic urticaria; CSU, chronic spontaneous urticaria, CIndU, chronic inducible urticaria, CSU + CIndU: Patients affected by both CSU and CIndU.

For CSU, sleep and mental status were reported by 54.9% and 46.9% of the physicians, respectively, as the most common affected areas. For CIndU, physical activities and mental status were reported by 65.5% and 29.2% of the physicians, respectively, as the most affected areas. For CSU + CIndU patients, sleep and mental status were reported by 50.4% and 40.3% of the physicians, respectively, as the most affected areas (Figure 1).

The most affected areas of a patient's life due to CSU, estimated by physicians, were sleep (mean: 3.0 ± 2.2), mental status (mean: 3.3 ± 2.2), and work/school (mean: 4.2 ± 2.1). The least affected areas were spare time (mean: 5.7 ± 2.0), eating behavior (mean: 5.2 ± 2.3), and appearance (mean: 5.0 ± 2.3) (*p* < 0.001 for all as compared to the reference, sleep). For CIndU, the most affected domains were physical activities (mean: 2.7 ± 2.3), mental status (mean: 3.9 ± 2.1), and appearance (mean: 4.5 ± 2.1). The greatest differences (relative to the reference, physical activities) were seen in eating behaviour (mean: 5.8 ± 2.2, *p* < 0.001), spare time (mean: 4.9 ± 2.1), and sleep (mean: 4.9 ± 2.4, *p* < 0.001) (Supplementary Table S2). As estimated by physicians, the median proportion of patients with severe CSU and CIndU who have experienced an impact on sleep, daily life, work, school, or leisure activities was 45.0% (IQR 25.0-60.0) and 30.0% (IQR 15.0-40.0), respectively.

### Estimation of CU prevalence and impact differed based on working at the UCARE, region, patient's age and specialty

Physicians working at UCARE (*n* = 183/232) vs. non-UCARE physicians (*n* = 49/232) differed in their estimation of CU prevalence in adults (median: 2.0% vs. 4.5%, *p* < 0.001) and children (median: 1.0% vs. 2.0%, *p* = 0.005), proportion of adult CSU patients among CU patients (70.0% vs. 60.5%, *p* = 0.027), and proportion of CSU patients with severe impact (50.0% vs. 30.0%, *p* = 0.042). UCARE experts also had significantly higher median confidence in estimating CU prevalence and burden as compared to other physicians (Table 2). Estimated adult CU prevalence differed by continent, with higher estimates reported by participants from Asia (*n* = 87/234) (median: 3.0%, IQR: 2.0–5.0) than those from Europe (*n* = 105/234) (median: 2.0%, IQR 1.0–4.3; *p* = 0.007). Compared to physicians who treat only adult patients (*n* = 56/232), those who treat both adults and pediatric patients (*n* = 160/232) showed significantly greater confidence in estimating the age of disease onset (*p* = 0.002), the percentages of CSU and CIndU in adult patients (*p* = 0.005), and the percentages of CU patients experiencing an impact on their sleep, daily activities, work, school, or leisure activities (*p* = 0.004) (Supplementary Table S4). Compared to non-urticaria-specialists [GPs, internists and pediatricians (*n* = 11/226)], allergists/immunologists (*n* = 126/226) and dermatologists (*n* = 89/226) showed significant higher confidence in estimating disease onset (*p* < 0.001 and *p* = 0.001), the percentages of CSU and CIndU in adult (allergists/immunologists *p* = 0.008) and pediatric patients (*p* = 0.001 and *p* = 0.029) and the percentages of CU patients experiencing an impact on their sleep, daily activities, work, school, or leisure activities (*p*=<0.001 and *p* = 0.007) (Supplementary Table S5). No significant differences were seen in CU prevalence estimation and burden based on respondent's age, clinical experience, place of work, or personal history of urticaria (Supplementary Tables S6–S10).

**Table 2:** Comparisons based on working at the UCARE.

UCARE		Yes (*n* = 183)	No (*n* = 49)	*p*-value independent-samples Mann–Whitney *U*-Test
Disease onset CSU (in years) median, (IQR), *n*		31.0, (28.0–40.0), 180	30.0, (23.8–40.0), 46	0.437
Disease onset CIndU (in years) median, (IQR), *n*		25.0, (20.0–30.0), 180	25.0, (20.0–30.0), 46	0.898
Disease onset CSU + CIndU (in years) median, (IQR), *n*		30.0, (25.0–35.0), 180	30.0, (25.0–35.0), 46	0.992
Confidence about disease onset median, (IQR), *n*		65.0, (45.0–76.0), 180	53.5, (32.0–70.3), 46	0.039
Estimation of prevalence of CU in adult patients (%) median, (IQR), *n*		2.0, (1.0–4.0), 172	4.5, (2.0–6.0), 44	<0.001
Percentage of CSU in CU adult patients median, (IQR), *n*		70.0, (60.0–80.0), 172	60.5, (40.0–75.0), 44	0.027
Percentage of concomitant CIndU in adults with CSU median, (IQR), *n*		30.0, (20.0–40.0), 172	34.5, (22.5–50.0), 44	0.103
Confidence about percentages for CSU and CIndU in adults median, (IQR), *n*		66.0, (50.0–78.0), 172	50.0, (37.0–68.8), 44	0.003
Estimation of prevalence of CU in pediatric patients (%) median, (IQR), *n*		1.0, (1.0–2.0), 139	2.0, (1.0–5.0), 31	0.005
Percentage of CSU in CU pediatric patients median, (IQR), *n*		62.0, (30.0–80.0), 139	50.0, (29.0–72.0), 31	0.578
Percentage of concomitant CIndU in pediatric patients with CSU median, (IQR), *n*		26.0, (11.0–47.0), 139	36.0, (10.0–50.0), 31	0.651
Confidence about percentages for CSU and CIndU in pediatric patients median, (IQR), *n*		60.0, (40.0–72.0), 139	50.0, (22.0–70.0), 31	0.138
Proportion of patients who have experienced severe impact on sleep, daily life, work, school or leisure activities median, (IQR), *n*	CSU	50.0, (30.0–60.0), 178	30.0, (20.0–50.0), 42	0.042
	CIndU	30.0 (13.8–40.0), 173	30.0, (20.0–40.0), 41	0.884
	CSU + CIndU	50.0, (30.0–70.0), 173	50.0, (30.0–60.0), 41	0.080
Confidence about percentages for proportion of CU patients have experienced an impact median, (IQR), *n*		69.0, (50.0–76.0), 178	59.0, (40.0–70.0), 42	0.032

UCARE, urticaria centers of reference and excellence; IQR, interquartile range; n, subgroup within the total group; CSU, chronic spontaneous urticaria; CIndU, chronic inducible urticaria; CSU + CIndU, Patients affected by both CSU and CIndU; CU, chronic urticaria.

## Discussion

This study evaluated physicians’ perceptions of prevalence and burden of CU. The results of this study are generally in line with other published data, although physicians working at specialized UCARE centers demonstrate a more realistic assessment regarding the prevalence of CU as well as a more accurate perception of the burden experienced by urticaria patients. Moreover, they reported greater confidence in their estimations compared to physicians not affiliated with UCARE centers. While the estimated prevalence and burden values align with previously published data, it is important to note that they reflect physicians’ perceptions rather than measured epidemiologic data.

The estimates in our study regarding the onset of CSU, CIndU, and CSU + CIndU in the same patient are largely consistent with figures reported in the literature, except for CSU where our study indicates a younger median age of onset: median 30.0 years (IQR 25.0–40.0) vs. literature reports of a median onset age of approximately 42 and 45 years (19, 20).

The estimation of the prevalence of CU among adult patients differed significantly depending on the continent of the physician surveyed. Physicians from Europe as compared to those from Asia estimated a significantly lower median prevalence of CU (2.0% vs. 3.0%). However, these findings align with previously published epidemiological data. Studies conducted in Europe report the point prevalence of CU in the population ranging approximately between 0.2% and 1.0%. In contrast, point prevalence estimates from Asian countries tend to be slightly higher, with values reported between 0.5% and 2.9% (4). This variation may reflect differences in environmental factors, genetic predispositions, or healthcare reporting practices between continents. The median estimated CU prevalence in pediatric patients in our study was 1.0% (IQR 1.0-3.0), which is in line with published data reporting a point prevalence between 0.73% and 1.97% (4).

The findings of this study highlight the substantial impact of CU—CSU and CIndU—on various domains of patients’ daily lives as perceived by treating physicians. Impaired sleep and mental well-being, as well as limitations in work or school activities, were identified as the most severely affected aspects in patients with CSU. These results are consistent with results of previous research showing that sleep impairment is a frequent and burdensome consequence of CSU, along with life impairment due to pruritus and looks (19, 21).

Physicians who treat both pediatric and adult patients with CU exhibit greater confidence than physicians who treat only adult patients specifically concerning the estimation of age of disease onset, the distribution of CSU and CIndU in adult patients, and the perceived impact of the disease on key areas of daily life (e.g., sleep, work, school, everyday activities, and leisure). A possible explanation lies in the broader clinical experience that comes with treating patients of different age groups, overall higher patient volume or specific practice setting.

Allergists/immunologists and dermatologists show significantly higher confidence compared to non-urticaria specialists (GPs, internists, and pediatricians) in all categories, likely because of their focused expertise on managing skin-related conditions including CU (22). To ensure accurate diagnosis and effective treatment of CU, increasing guideline awareness among non-specialist physicians is essential (23).

The significant differences in the estimation of the proportion of patients who have experienced severe impact on sleep, daily life, work, school, or leisure activities due to CSU between physicians working at a UCARE center vs. those who don’t point to the underestimated burden of CSU in a subset of respondents. These findings align with the survey conducted by Mosnaim et al., who found that physicians often underestimate and underreport the severity of CSU in their patients (21). In general, our results show that the estimations made by physicians working at UCARE centers tend to align more with results reported in the published literature and are associated with higher confidence. This may, in part, be explained by the fact that working in specialized urticaria centers includes increased physician interactions with urticaria patients and/or focused education and awareness of urticaria-related guidelines and published data that leads to a more accurate physician assessment.

Although not statistically significant, non-urticaria-specialists tended to estimate a lower proportion of patients experiencing a severe impact on daily life due to CSU, CIndU and CSU + CIndU, which may suggest limited awareness and an underestimation of disease burden. Limited awareness may contribute to an underestimation of the disease's impact on patients’ quality of life. This can result in suboptimal treatment, as a physician who underestimates the severity of the disease may choose a less effective treatment option, which can lead to a poorer treatment outcome as shown by Wolfenden et al. in the treatment of asthma (24). For patients, not feeling understood and taken seriously can lead to increased frustration, lower treatment adherence and therefore delayed achievement of disease control (25). Open communication, a patient centered approach, and shared decision making are crucial parts of the relationship between a patient and physician (26). A treatment tailored to the patient's needs can lead to better disease control and an improved quality of life.

There are strengths and limitations to this study. One strength of this study is its international scope, including participants from 46 countries. This provides a previously unavailable global perspective on how physicians assess CU, although the data may not be generalizable to all continents, especially those under-represented such as north America, Africa and Oceania. An analysis of the participants in our study shows that the majority (68.1%) work at university/training hospitals, of whom 78.7% are affiliated with a UCARE center. To gain a more differentiated understanding of physicians’ perspectives on CU, it would be beneficial to conduct further studies focusing more on GPs and other non-UCARE physicians, as they are often the treating physician for patients with CU (8, 14).

Due to the small number of participants in the non-specialist (*n* = 11) and pediatric patients only (*n* = 10) groups, these subgroup analyses were underpowered, and the corresponding results should therefore be interpreted with caution and as preliminary observations rather than definitive results. The reported prevalence and burden values represent physicians’ subjective estimations which may be influenced by recall bias and do not constitute direct epidemiologic measurements. This distinction is essential to avoid overinterpretation of the results. The indirect limitation is a lack of literature data on the impairment of specific areas of daily life caused by CSU + CIndU in the same patient. Further studies in this area would allow to more accurately capture the burden experienced by patients and to better tailor treatment strategies and support services accordingly.

## Conclusion

The results of our multicenter, cross-sectional study highlight the need for enhancing awareness of non-UCARE physicians regarding the prevalence and burden of CU. Targeted initiatives may include dissemination of clinical guidelines, educational webinars explaining referral indications, need for shared decision making and workshops on the use of patient reported outcome measurements. Improved physician knowledge of CU and awareness of disease burden may, ultimately, lead to improved CU patient care and an increased likelihood of achieving complete disease control, the main goal of urticaria treatment (27)*.*

## Data Availability

The raw data supporting the conclusions of this article will be made available by the authors, upon reasonable request.
