# Supplementary material for: Physician estimation of the prevalence and clinical impact of chronic urticaria: results of the global, multicenter UCARE CU-PAPER study
Source: Front Allergy. 2026 Jan 12;6:1732893. doi: 10.3389/falgy.2025.1732893 (PMC12833087; doi:10.3389/falgy.2025.1732893)
Supplement: Supplementary file 1 [file Datasheet1.docx]

Supplementary Material

**SUPPLEMENT TABLES**

**Suppl. Table S1. Demographic data**

| **Parameter** | | **Results** |
| --- | --- | --- |
| Total number of people who filled in the questionnaire until a stop command (did not agree to participate, not a physician, don’t treat patients with CU etc.) or finished it completely | | 244 |
| agreed to participate % (n) | | 97.5 (238) |
| did not agree to participate % (n) | | 2.5 (6) |
| Region % (n) | Number of physicians who indicated a region of residence | 234 |
|  | Africa | 1.7 (4) |
|  | Asia | 37.2 (87) |
|  | Europe | 44.9 (105) |
|  | North America | 3.8 (9) |
|  | Oceania | 0 (0) |
|  | South America | 12.4 (29) |
| Country % (n) | Number of physicians who indicated a country of residence | 234 |
|  | United Arab Emirates | 0.9 (2) |
|  | Argentina | 1.7 (4) |
|  | Austria | 0.4 (1) |
|  | Bulgaria | 1.3 (3) |
|  | Brazil | 7.8 (18) |
|  | Canada | 0.9 (2) |
|  | China | 0.9 (2) |
|  | Colombia | 1.7 (4) |
|  | Germany | 15.4 (36) |
|  | Denmark | 1.7 (4) |
|  | Algeria | 0.4 (1) |
|  | Ecuador | 1.3 (3) |
|  | Spain | 2.6 (6) |
|  | France | 2.6 (6) |
|  | UK | 1.7 (4) |
|  | Georgia | 6.4 (15) |
|  | Greece | 1.3 (3) |
|  | China (Hong Kong S.A.R.) | 0.9 (2) |
|  | Croatia | 0.9 (2) |
|  | Ireland | 0.9 (2) |
|  | Israel | 0.4 (1) |
|  | India | 1.3 (3) |
|  | Iran (Islamic Republic of) | 2.1 (5) |
|  | Italy | 2.1 (5) |
|  | Japan | 1.7 (4) |
|  | Kuwait | 0.9 (2) |
|  | Kazakhstan | 3.0 (7) |
|  | Lithuania | 0.4 (1) |
|  | Macedonia | 0.4 (1) |
|  | Mexico | 0.9 (2) |
|  | Malaysia | 0.4 (1) |
|  | Netherlands | 0.9 (2) |
|  | Oman | 0.9 (2) |
|  | Peru | 0.4 (1) |
|  | Philippines | 2.1 (5) |
|  | Poland | 3.0 (7) |
|  | Portugal | 2.1 (5) |
|  | Qatar | 0.4 (1) |
|  | Russian Federation | 1.7 (4) |
|  | Saudi Arabia | 0.9 (2) |
|  | Slovenia | 0.9 (2) |
|  | Thailand | 1.7 (4) |
|  | Turkey | 13.3 (31) |
|  | Taiwan | 3.9 (9) |
|  | USA | 1.7 (4) |
|  | South Africa | 1.3 (3) |
| Gender, % (n/N) female | | 54.3 (127/234) |
| Age in years Median, (IQR), N | | 48.5, (38.3-58), 220 |
| Age groups in years, % (n) | 25-35 | 16.8 (37) |
|  | 36-50 | 37.7 (83) |
|  | 51-65 | 39.6 (87) |
|  | 66 or older | 5.9 (13) |
| Specialty, % (n) | Total number of replies | 234 |
|  | Allergy/Allergology or Allergy/clinical Immunology | 54.7 (128) |
|  | Dermatology | 38.9 (91) |
|  | General Practice/Primary Care | 4.3 (10) |
|  | Internal Medicine | 1.3 (3) |
|  | Pediatrics | 0.9 (2) |
| Clinical practice, % (n/N) yes  % (n/N) no | | 99.1 (232/234)  0.9 (2/234) |
| Years in clinical practice Median, (IQR), N | | 20.0, (10.0-30.0), 232 |
| Primary place of work, % (n) | Total number of replies | 232 |
|  | University/Training hospital | 68.1 (158) |
|  | Public hospital | 9.1 (21) |
|  | Private hospital | 7.3 (17) |
|  | Private practice (office) | 13.4 (31) |
|  | Other (family physician/private clinic/outpatient clinic/ pharmaceutical Industry/residency program) | 2.2 (5) |
| UCARE % (n/N) yes | | 78.9 (183/232) |

Abbreviations: UCARE: Urticaria Centers of Reference and Excellence, IQR: interquartile range, N: Total group size, n: subgroup within the total group, CU: chronic urticaria, S.A.R.: Special Administrative Region, UK: United Kingdom, USA: United States of America.

**Suppl. Table S2. Estimation of CU prevalence and burden**

| Do you see patients with CU, % (n) | Total number of replies | | 232 |
| --- | --- | --- | --- |
|  | No | | 2.6 (6) |
|  | Only adult patients | | 24.1 (56) |
|  | Only pediatric patients | | 4.3 (10) |
|  | Both adult and pediatric patients | | 69.0 (160) |
| Disease onset CSU (years), Median, (IQR) | | | 30.0, (25.0-40.0) |
| Disease onset CIndU (years), Median, (IQR) | | | 25.0, (20.0-30.0) |
| Disease onset CSU+CIndU (years), Median, (IQR) | | | 30.0, (25.0-35.0) |
| Confidence about disease onset, Median, (IQR) | | | 60.0, (40.0-75.0) |
| CU estimated prevalence in adults, Median, (IQR) | | | 2.5, (1.0-5.0) |
| Percentage of CSU in CU adult patients, Median, (IQR) | | | 70.0, (55.0-80.0) |
| Percentage of concomitant CIndU in adults with CSU, Median, (IQR) | | | 30.0, (20.0-45.0) |
| Confidence about percentages for CSU and CIndU in adults, Median, (IQR) | | | 64.0, (50.0-75.0) |
| CU estimated prevalence in pediatric patients, Median, (IQR) | | | 1.0, (1.0-3.0) |
| Percentage of CSU in CU in pediatric patients, Median, (IQR) | | | 60.0, (29.8-78.5) |
| Percentage of concomitant CIndU in pediatric patients with CSU, Median, (IQR) | | | 29.5, (11.0-50.0) |
| Confidence about percentages for CSU and CIndU in pediatric patients, Median, (IQR) | | | 57.5, (32.0-71.0) |
| most affected aspects of patient’s life due to CSU | Physical activities, mean (SD) | | 4.9 (2.0) |
|  | Sleep, mean (SD) | | 3.0 (2.2) |
|  | Sleep  Ranking, % (n) | 1 (most affected) | 36.7 (83) |
|  |  | 2 | 18.1 (41) |
|  |  | 3 | 12.4 (28) |
|  |  | 4 | 9.3 (21) |
|  |  | 5 | 6.6 (15) |
|  |  | 6 | 6.2 (14) |
|  |  | 7 | 6.6 (15) |
|  |  | 8 (least affected) | 4.0 (9) |
|  | Spare time, mean (SD) | | 5.7 (2.0) |
|  | Social relationships, mean (SD) | | 4.7 (1.8) |
|  | Eating behavior, mean (SD) | | 5.2 (2.3) |
|  | Mental status, mean (SD) | | 3.3 (2.2) |
|  | Mental status ranking, % (n) | 1 (most affected) | 26.1 (59) |
|  |  | 2 | 20.8 (47) |
|  |  | 3 | 16.8 (38) |
|  |  | 4 | 4.9 (11) |
|  |  | 5 | 11.5 (26) |
|  |  | 6 | 7.1 (16) |
|  |  | 7 | 7.5 (17) |
|  |  | 8 (least affected) | 5.3 (12) |
|  | Appearance, mean (SD) | | 5.0 (2.3) |
|  | Work/School, mean (SD) | | 4.2 (2.1) |
|  | Work/school Ranking, % (n) | 1 (most affected) | 10.7 (24) |
|  |  | 2 | 14.6 (33) |
|  |  | 3 | 19.0 (43) |
|  |  | 4 | 15.5 (35) |
|  |  | 5 | 9.7 (22) |
|  |  | 6 | 12.4 (28) |
|  |  | 7 | 10.2 (23) |
|  |  | 8 (least affected) | 8.0 (18) |
| most affected aspects of patient’s life due to CIndU | Physical activities, mean (SD) | | 2.7 (2.3) |
|  | Physical activities ranking, % (n) | 1 (most affected) | 51.3 (116) |
|  |  | 2 | 14.2 (32) |
|  |  | 3 | 8.0 (18) |
|  |  | 4 | 5.8 (13) |
|  |  | 5 | 4.0 (9) |
|  |  | 6 | 5.8 (13) |
|  |  | 7 | 3.5 (8) |
|  |  | 8 (least affected) | 7.5 (17) |
|  | Sleep, mean (SD) | | 4.9 (2.4) |
|  | Spare time, mean (SD) | | 4.9 (2.1) |
|  | Social relationships, mean (SD) | | 4.8 (1.5) |
|  | Eating behavior, mean (SD) | | 5.8 (2.3) |
|  | Mental status, mean (SD) | | 3.9 (2.1) |
|  | Mental status ranking, % (n) | 1 (most affected) | 13.3 (30) |
|  |  | 2 | 15.9 (36) |
|  |  | 3 | 17.3 (39) |
|  |  | 4 | 19.0 (43) |
|  |  | 5 | 9.7 (22) |
|  |  | 6 | 10.2 (23) |
|  |  | 7 | 9.3 (21) |
|  |  | 8 (least affected) | 5.3 (12) |
|  | Appearance, mean (SD) | | 4.5 (2.1) |
|  | Appearance ranking, % (n) | 1 (most affected) | 8.4 (19) |
|  |  | 2 | 13.7 (31) |
|  |  | 3 | 15.0 (34) |
|  |  | 4 | 15.0 (34) |
|  |  | 5 | 8.0 (18) |
|  |  | 6 | 16.4 (37) |
|  |  | 7 | 16.4 (37) |
|  |  | 8 (least affected) | 7.1 (16) |
|  | Work/School, mean (SD) | | 4.6 (2.2) |
| most affected aspects of patient’s life due to CSU+CIndU | Physical activities, mean (SD) | | 3.7 (2.1) |
|  | Physical activities ranking, % (n) | 1 (most affected) | 15.9 (36) |
|  |  | 2 | 20.8 (47) |
|  |  | 3 | 15.0 (34) |
|  |  | 4 | 12.8 (29) |
|  |  | 5 | 10.2 (23) |
|  |  | 6 | 12.4 (28) |
|  |  | 7 | 7.5 (17) |
|  |  | 8 (least affected) | 5.3 (12) |
|  | Sleep, mean (SD) | | 3.3 (2.3) |
|  | Sleep ranking, % (n) | 1 (most affected) | 28.3 (64) |
|  |  | 2 | 22.1 (50) |
|  |  | 3 | 12.0 (27) |
|  |  | 4 | 8.9 (20) |
|  |  | 5 | 9.3 (21) |
|  |  | 6 | 5.3 (12) |
|  |  | 7 | 8.0 (18) |
|  |  | 8 (least affected) | 6.2 (14) |
|  | Spare time, mean (SD) | | 5.6 (1.9) |
|  | Social relationships, mean (SD) | | 4.9 (1.7) |
|  | Eating behavior, mean (SD) | | 5.7 (2.3) |
|  | Mental status, mean (SD) | | 3.6 (2.2) |
|  | Mental status ranking, % (n) | 1 (most affected) | 23.5 (53) |
|  |  | 2 | 16.8 (38) |
|  |  | 3 | 15.9 (36) |
|  |  | 4 | 11.1 (25) |
|  |  | 5 | 9.7 (22) |
|  |  | 6 | 9.7 (22) |
|  |  | 7 | 6.6 (15) |
|  |  | 8 (least affected | 6.6 (15) |
|  | Appearance, mean (SD) | | 4.8 (2.3) |
|  | Work/School, mean (SD) | | 4.4 (2.2) |
| Proportion of CSU patients which have experienced an impact on sleep, daily life, work, school or leisure activities, Median, (IQR) | Valid responses | | 220 |
|  | Severe CSU | | 45.0, (25.0-60.0) |
|  | Moderate CSU | | 30.0, (30.0-50.0) |
|  | Mild CSU | | 20.0, (10.0-30.0) |
| Proportion of CIndU patients which have experienced an impact on sleep, daily life, work, school or leisure activities, Median, (IQR) | Valid responses | | 214 |
|  | Severe CIndU | | 30.0, (15.0-40.0) |
|  | Moderate CIndU | | 40.0, (30.0-50.0) |
|  | Mild CIndU | | 30.0, (20.0-40.0) |
| Proportion of CSU+CIndU patients which have experienced an impact on sleep, daily life, work, school or leisure activities, Median, (IQR) | Valid responses | | 214 |
|  | Severe CSU+CIndU | | 50.0, (30.0-65.0) |
|  | Moderate CSU+CIndU | | 30.0, (20.0-45.0) |
|  | Mild CSU+CIndU | | 10.0, (10.0-20.0) |
| Confidence in the estimated impact on sleep, daily life, work, school or leisure activities Median, (IQR), N | | | 65.0, (50.0-75.0), 220 |

Abbreviations: IQR: interquartile range, N: Total group size, n: subgroup within the total group, SD: standard deviation, CSU: chronic spontaneous urticaria, CIndU: chronic inducible urticaria, CSU+CIndU: Patients affected by both CSU and CIndU, CU: chronic urticaria

**Suppl. Table S3. Participants affected by urticaria**

| AU personally affected % (n/N) yes | | 52.2 (118/226) |
| --- | --- | --- |
| CU personally affected % (n/N) yes | | 15.5 (35/226) |
| CU personally affected Type, % (n/N) | Total responses | 35 |
|  | Isolated CSU | 40.0 (14/35) |
|  | Isolated CIndU | 37.1 (13/35) |
|  | CSU+CIndU | 22.9 (8/35) |
| CU personally affected years, Median, (IQR) | | 5.0, (2.0-10.0) |
| Activity of disease, % (n/N) | Total responses | 35 |
|  | High | 14.3 (5/35) |
|  | Moderate | 28.6 (10/35) |
|  | Low | 57.1 (20/35) |
| Most frequent symptoms, % (n/N) | Itch | 88.6 (31/35) |
|  | Wheals | 80.0 (28/35) |
|  | Angioedema | 28.6 (10/35) |
|  | Others: Sleep disturbances, change in skin color, medication side effects including dry eyes and mouth | 2.9 (1/35) |
| Most bothersome symptom, % (n/N) | Number of responses | 35 |
|  | Itch | 65.7 (23/35) |
|  | Wheals | 14.3 (5/35) |
|  | Angioedema | 20.0 (7/35) |

Abbreviations: IQR: interquartile range, N: Total group size, n: subgroup within the total group, CSU: chronic spontaneous urticaria, CIndU: chronic inducible urticaria, CSU+CIndU: Patients affected by both CSU and CIndU, CU: chronic urticaria, AU: acute urticaria, Meds: Medicaments

**Suppl. Table S4. Comparisons based on patient age**

| **Patient clientele** | | **Adult patients (n=56)**  **(Group 1)** | **Pediatric patients (n=10)**  **(Group 2)** | **Both adults and pediatric patients (n=160)**  **(Group 3)** | **p-value** Kruskal Wallis H/ independent-Samples Mann-Whitney U Test |
| --- | --- | --- | --- | --- | --- |
| **Disease onset CSU (in years) Median, (IQR), n** | | 31.0, (30.0-40.0), 56 | 19.5, (9.8-31.3), 10 | 30.0, (25.0-40.0), 160 | 0.060 |
| **Disease onset CIndU (in years) Median, (IQR), n** | | 30.0, (23.5-30.0), 56 | 13.5, (8.0-22.5), 10 | 25.0, (20.0-30.0), 160 | 0.001  G1 vs G2 (0.001)  G2 vs. G3 (0.040)  G1 vs. G3 (0.021) |
| **Disease onset CSU+CIndU (in years) Median, (IQR), n** | | 30.0, (28.0-35.0), 56 | 18.0, (11.5-41.3), 10 | 30.0, (25.0-35.0), 160 | 0.105 |
| **Confidence about disease onset Median, (IQR) n** | | 50.0, (35.0-68.0), 56 | 41.0, (23.8-74.3), 10 | 66.5, (50.0-80.0), 160 | 0.002  G1 vs. G3 (0.002) |
| **Estimation of prevalence of CU in adult patients (%) Median, (IQR), n** | | 3.0, (2.0-5.0), 56 | **-** | 2.0, (1.0-4.0), 160 | 0.150 |
| **Percentage of CSU in CU adult patients Median, (IQR), n** | | 70.0, (60.0-80.0), 56 | **-** | 70.0, (51.0-80.0), 160 | 0.667 |
| **Percentage of concomitant CIndU in adults with CSU Median, (IQR), n** | | 25.0, (20.0-41.8), 56 | **-** | 30.0, (20.0-48.3), 160 | 0.357 |
| **Confidence about percentages for CSU and CIndU in adults Median, (IQR), n** | | 50.0, (33.3-70.0), 56 | **-** | 65.5, (50.0-80.0), 160 | 0.005 |
| **Estimation of prevalence of CU in pediatric patients (%) Median, (IQR), n** | | **-** | 2.5, (1.0-5.0), 10 | 1.0, (1.0-3.0), 160 | 0.113 |
| **Percentage of CSU in CU pediatric patients Median, (IQR), n** | | **-** | 71.5, (49.3-90.0), 10 | 60.0, (29.3-76.5), 160 | 0.138 |
| **Percentage of concomitant CIndU in pediatric patients with CSU Median, (IQR), n** | | **-** | 10.0, (4.5-55.3), 10 | 30.0, (13.0-50.0), 160 | 0.214 |
| **Confidence about percentages for CSU and CIndU in pediatric patients Median, (IQR), n** | | **-** | 70.5, (33.5-77.0), 10 | 53.5 (32.0-70.8), 160 | 0.401 |
| **Proportion of patients** **who** **have experienced severe impact on sleep, daily life, work, school or leisure activities Median, (IQR), n** | **CSU** | 40.0, (30.0-50.0), 55 | 40.0, (16.3-57.5), 10 | 50.0, (25.0-60.0), 155 | 0.362 |
|  | **CIndU** | 25.0, (10.0-34.0), 53 | 22.5, (10.0-36.3), 10 | 30.0, (20.0-40.0), 151 | 0.240 |
|  | **CSU+CIndU** | 40.0, (30.0-60.0), 54 | 47.5, (21.3-63.8), 9 | 50.0, (35.0-70.0), 151 | 0.076 |
| **Confidence about percentages for proportion of CU patients have experienced an impact Median, (IQR), n** | | 50.0, (40.0-70.5), 55 | 66.5, (41.0-75.8), 10 | 70.0, (54.0-77.0), 155 | 0.004  G1 vs. G3 (0.003) |

Abbreviations: IQR: interquartile range, n: subgroup within the total group, CSU: chronic spontaneous urticaria, CIndU: chronic inducible urticaria, CSU+CIndU: Patients affected by both CSU and CIndU, CU: chronic urticaria

**Suppl. Table S5.** **Comparisons based on specialty**

| **Specialty** | | **Allergy/Immunology (n=126)**  **(Group 1)** | **Dermatology (n=89)**  **(Group 2)** | **Non-specialists (GPs, internal medicine, Pediatrics) (n=11)**  **(Group 3)** | **p-value** Kruskal Wallis H Test |
| --- | --- | --- | --- | --- | --- |
| **Disease onset CSU (in years) Median, (IQR), n** | | 30.0, (27.3-38.5), 126 | 35.0, (26.5-40.0), 89 | 30.0, (16.0-35.0), 11 | 0.214 |
| **Disease onset CIndU (in years) Median, (IQR), n** | | 25.0, (20.0-30.0), 126 | 25.0, (20.0-30.0), 89 | 25.0, (10.0-30.0), 11 | 0.579 |
| **Disease onset CSU+CIndU (in years) Median, (IQR), n** | | 30.0, (25.0-35.0), 126 | 30.0, (25.0-35.0), 89 | 30.0, (20.0-45.0), 11 | 0.620 |
| **Confidence about disease onset Median, (IQR), n** | | 68.0, (50.0-80.0), 126 | 60.0, (35.0-70.0), 89 | 27.0, (17.0-35.0), 11 | <0.001  G1 vs. G3 <0.001  G2 vs. G3 0.001 |
| **Estimation of prevalence of CU in adult patients (%) Median, (IQR), n** | | 3.0, (1.0-5.0), 118 | 2.0, (1.0-4.0), 89 | 5.0, (2.5-7.5), 9 | 0.012  G2 vs. G3 0.041 |
| **Percentage of CSU in CU adult patients Median, (IQR), n** | | 70.0, (51.0-80.0), 118 | 70.0, (60.0-80.0), 89 | 40.0, (32.5-74.0), 9 | 0.203 |
| **Percentage of concomitant CIndU in adults with CSU Median, (IQR), n** | | 30.0, (20.0-45.0), 118 | 30.0, (20.0-40.5), 89 | 50.0 (28.5-62.5), 9 | 0.058 |
| **Confidence about percentages for CSU and CIndU in adults Median, (IQR), n** | | 65.5, (50.0-78.3), 118 | 60.0, (42.0-71.5), 89 | 40.0, (10.5-52.0), 9 | 0.008  G1 vs. G3 0.012 |
| **Estimation of prevalence of CU in pediatric patients (%) Median, (IQR), n** | | 1.0, (1.0-3.0), 92 | 1.0, (1.0-2.0), 73 | 3.0, (1.5-6.5), 5 | 0.092 |
| **Percentage of CSU in CU pediatric patients Median, (IQR), n** | | 60.0, (20.0-74.8), 92 | 60.0, (32.5-80.0), 73 | 75.0, (33.0-91.0), 5 | 0.441 |
| **Percentage of concomitant CIndU in pediatric patients with CSU Median, (IQR), n** | | 30.0, (10.0-45.0), 92 | 29.0, (15.0-50.0), 73 | 20.0, (6.5-57.5), 5 | 0.601 |
| **Confidence about percentages for CSU and CIndU in pediatric patients Median, (IQR), n** | | 62.0, (50.0-77.0), 92 | 50.0, (30.0-65.5), 73 | 10.0, (5.5-27.5), 5 | <0.001  G1 vs. G2 0.017  G1 vs. G3 0.001  G2 vs. G3 0.029 |
| **Proportion of patients** **who** **have experienced severe impact on sleep, daily life, work, school or leisure activities Median, (IQR), n** | **CSU** | 40.0, (26.3-50.0), 116 | 50.0, (25.0-60.0), 83 | 30.0, (20.0-50.0), 10 | 0.094 |
|  | **CIndU** | 25.0, (15.0-40.0), 116 | 30.0, (15.0-40.0), 83 | 20.0, (10.0-30.0), 10 | 0.113 |
|  | **CSU+**  **CIndU** | 50.0, (30.0-60.0), 116 | 50.0, (40.0-70.0), 83 | 35.0, (20.0-50.0), 10 | 0.114 |
| **Confidence about percentages for proportion of CU patients have experienced an impact Median, (IQR), n** | | 70.0, (52.5-75.8), 116 | 63.0, (50.0-75.0), 83 | 30.0, (19.8-61.8), 10 | <0.001  G1 vs. G3 <0.001  G2 vs. G3 0.007 |

Abbreviations: IQR: interquartile range, n: subgroup within the total group, CSU: chronic spontaneous urticaria, CIndU: chronic inducible urticaria, CSU+CIndU: Patients affected by both CSU and CIndU, CU: chronic urticaria

**Suppl. Table S6. Comparisons based on age groups**

| **Age groups** | | **25-35 years (n=37)**  **(Group 1)** | **36-50 years (n=83)**  **(Group 2)** | **51-65 years (n=87)**  **(Group 3)** | **66 years or older (n=13)**  **(Group 4)** | **p-value** Kruskal Wallis H Test |
| --- | --- | --- | --- | --- | --- | --- |
| **Disease onset CSU (in years) Median, (IQR), n** | | 34.5, (25.0-40.0), 36 | 30.0, (25.0-40.0), 79 | 30.0, (27.3-40.0), 86 | 35.0, (30.0-38.8), 12 | 0.519 |
| **Disease onset CIndU (in years) Median, (IQR), n** | | 30.0, (24.3-33.8), 36 | 25.0, (20.0-30.0), 79 | 25.0, (20.0-28.0), 86 | 30.0, (25.0-35.8), 12 | 0.001  G1 vs. G3 (0.017).  G3 vs. G4 (0.029) |
| **Disease onset CSU+CIndU (in years) Median, (IQR), n** | | 30.0, (25.8-40.0,) 36 | 30.0, (25.0-35.0), 79 | 30.0, (25.0-35.0), 86 | 30.0, (30.0-38.8), 12 | 0.268 |
| **Confidence about disease onset Median, (IQR), n** | | 50.0, (31.0-70.5), 36 | 68.0, (50.0-80.0), 79 | 60.0, (40.0-75.0), 86 | 58.0, (42.5-73.8), 12 | 0.079 |
| **Estimation of prevalence of CU in adult patients (%) Median, (IQR), n** | | 3.0, (2.0-4.5), 33 | 2.5, (1.0-5.0), 76 | 2.0, (1.0-5.0), 82 | 2.0, (1.3-3.0), 12 | 0.649 |
| **Percentage of CSU in CU adult patients, Median, (IQR), n** | | 65.0, (45.0-79.5), 33 | 70.0, (60.0-80.0), 76 | 70.0, (50.0-80.0), 82 | 74.5, (65.3-80.8), 12 | 0.496 |
| **Percentage of concomitant CIndU in adults with CSU, Median, (IQR), n** | | 25.0, (20.0-40.0), 33 | 30.0, (20.0-40.0), 76 | 30.5, (20.0-60.0), 82 | 41.5, (13.0-53.8), 12 | 0.172 |
| **Confidence about percentages for CSU and CIndU in adults, Median, (IQR), n** | | 54.0, (30.0-73.0), 33 | 65.0, (41.0-75.0), 76 | 63.0, (50.0-75.0), 82 | 70.0, (52.5-78.8), 12 | 0.298 |
| **Estimation of prevalence of CU in pediatric patients (%) Median, (IQR), n** | | 1.5, (1.0-3.8), 20 | 1.0, (1.0-3.0), 62 | 1.0, (1.0-3.0), 68 | 1.0, (1.0-1.5), 9 | 0.414 |
| **Percentage of CSU in CU pediatric patients, Median, (IQR), n** | | 50.0, (20.0-71.5), 20 | 65.0, (34.8-80.0), 62 | 50.0, (22.0-73.8), 68 | 70.0, (60.0-85.0), 9 | 0.074 |
| **Percentage of concomitant CIndU in pediatric patients with CSU, Median, (IQR), n** | | 40.0, (10.0-67.5), 20 | 30.0, (12.8-48.5), 62 | 26.0, (10.0-48.3), 68 | 25.0, (10.0- 40.0), 9 | 0.470 |
| **Confidence about percentages for CSU and CIndU in pediatric patients, Median, (IQR), n** | | 50.0, (32.5-65.5), 20 | 59.5, (35.8-73.5), 62 | 50.0, (30.0-71.0), 68 | 60.0, (50.0-71.0), 9 | 0.824 |
| **Proportion of patients** **who** **have experienced severe impact on sleep, daily life, work, school or leisure activities Median, (IQR), n** | **CSU** | 50.0, (30.0-50.0), 35 | 40.0, (20.0-50.0), 77 | 50.0, (30.0-60.0), 85 | 27.5, (15.0-42.5), 12 | 0.017 no significance after adjustment |
|  | **CIndU** | 30.0, (10.0-40.0), 34 | 30.0, (12.5-40.0), 76 | 30.0, (20.0-40.0), 82 | 20.0, (18.8-36.3), 11 | 0.976 |
|  | **CSU+CIndU** | 50.0, (35.0-60.0), 34 | 50.0, (30.0-60.0), 75 | 50.0, (40.0-70.0), 83 | 41.5 (25.0-62.5), 11 | 0.355 |
| **Confidence about percentages for proportion of CU patients have experienced an impact Median, (IQR), n** | | 50.0, (39.5-70.0), 35 | 65.5 (50.0-75.0), 77 | 68.5, (50.0-76.8), 85 | 67.0, (62.0-75.0), 12 | 0.017  G1 vs. G3 (0.026) |

Abbreviations: IQR: interquartile range, n: subgroup within the total group, CSU: chronic spontaneous urticaria, CIndU: chronic inducible urticaria, CSU+CIndU: Patients affected by both CSU and CIndU, CU: chronic urticaria

**Suppl. Table S7. Comparisons based on clinical experience**

| **Years of clinical experience** | | **Under 10 years in clinical practice (n=47)**  **(Group 1)** | **10-25 years in clinical practice (n=104)**  **(Group 2)** | **More than 25 years in clinical practice (n=81)**  **(Group 3)** | **p-value** Kruskal Wallis H Test |
| --- | --- | --- | --- | --- | --- |
| **Disease onset CSU (in years) Median, (IQR), n** | | 30.0, (20.0-40.0), 42 | 30.0, (25.0-39.3), 103 | 30.0, (30.0-40.0), 81 | 0.735 |
| **Disease onset CIndU (in years) Median, (IQR), n** | | 30.0, (20.0-32.5), 42 | 25.0, (20.0-30.0), 103 | 25.0, (20.0-28.0), 81 | 0.014  G1 vs. G3 (0.011) |
| **Disease onset CSU+CIndU (in years) Median, (IQR), n** | | 30.0, (22.5-39.0), 42 | 30.0, (25.0-35.0), 103 | 30.0, (25.0-31.5), 81 | 0.593 |
| **Confidence about disease onset Median, (IQR), n** | | 50.0, (34.0-75.5), 42 | 66.0, (50.0-80.0), 103 | 60.0, (50.0-76.0), 81 | 0.055 |
| **Estimation of prevalence of CU in adult patients (%) Median, (IQR), n** | | 3.0, (2.0-4.0), 39 | 2.0, (1.0-5.0), 99 | 2.0, (1.0-4.0), 78 | 0.825 |
| **Percentage of CSU in CU adult patients Median, (IQR), n** | | 70.0, (40.0-80.5), 39 | 70.0, (60.0-80.0), 99 | 70.0, (50.0-80.0), 78 | 0.658 |
| **Percentage of concomitant CIndU in adults with CSU Median, (IQR), n** | | 30.0, (15.5-50.0), 39 | 30.0, (20.0-42.0), 99 | 31.5, (20.0-48.0), 78 | 0.379 |
| **Confidence about percentages for CSU and CIndU in adults Median, (IQR), n** | | 60.0, (46.5-80.5), 39 | 64.0, (50.0-75.0), 99 | 65.5, (47.0-80.0), 78 | 0.679 |
| **Estimation of prevalence of CU in pediatric patients (%) Median, (IQR), n** | | 2.0, (1.0-3.0), 28 | 1.0, (1.0-3.0), 82 | 1.0, (1.0-3.0), 60 | 0.384 |
| **Percentage of CSU in CU pediatric patients Median, (IQR), n** | | 59.5, (29.5-78-5), 28 | 65.5 (23.8-80.0), 82 | 50.0, (30.0-70.0), 60 | 0.812 |
| **Percentage of concomitant CIndU in pediatric patients with CSU Median, (IQR), n** | | 39.0, (15.0-55.0), 28 | 25.5, (10.8-40.8), 82 | 28.5, (15.0-50.0), 60 | 0.320 |
| **Confidence about percentages for CSU and CIndU in pediatric patients Median, (IQR), n** | | 60.5, (25.0-76.0), 28 | 54.0, (39.3-70.0), 82 | 53.5, (31.0-71.5), 60 | 0.903 |
| **Proportion of patients** **who** **have experienced severe impact on sleep, daily life, work, school or leisure activities Median, (IQR), n** | **CSU** | 50.0, (30.0-50.0), 41 | 40.0, (20.0-60.0), 99 | 50.0, (25.0-60.0), 80 | 0.715 |
|  | **CIndU** | 30.0, (20.0-40.0), 41 | 30.0, (10.0-40.0), 95 | 30.0, (20.0-40.0), 78 | 0.180 |
|  | **CSU+CIndU** | 50.0, (37.5-60.0), 39 | 50.0, (30.0-60.0), 97 | 50.0, (30.0-70.0), 78 | 0.663 |
| **Confidence about percentages for proportion of CU patients have experienced an impact Median, (IQR), n** | | 59.0, (40.0-70.5), 41 | 66.0, (50.0-74.5), 99 | 70.0, (50.0-80.0), 80 | 0.045  G1 vs. G3 (0.042) |

Abbreviations: IQR: interquartile range, n: subgroup within the total group, CSU: chronic spontaneous urticaria, CIndU: chronic inducible urticaria, CSU+CIndU: Patients affected by both CSU and CIndU, CU: chronic urticaria

**Suppl. Table S8. Comparisons based on place of work**

| **Place of work** | | **University hospital/Training hospital (n=158)**  **(Group 1)** | **Public hospital (n=21)**  **(Group 2)** | **Private hospital (n=17)**  **(Group 3)** | **Private practice (office) (n=31)**  **(Group 4)** | **p-value** Kruskal Wallis H Test |
| --- | --- | --- | --- | --- | --- | --- |
| **Disease onset CSU (in years) Median, (IQR), n** | | 34.0, (27.3-40.0), 156 | 30.0, (26.3-43.8), 21 | 30.0, (18.0-36.5), 17 | 30.0, (23.8-40.5), 28 | 0.208 |
| **Disease onset CIndU (in years) Median, (IQR), n** | | 25.0, (20.0-30.0), 156 | 22.5, (18.5-25.0), 21 | 25.0, (16.0-27.5), 17 | 25.0, (19.5-30.0), 28 | 0.587 |
| **Disease onset CSU+CIndU (in years) Median, (IQR), n** | | 30.0, (25.0-35.0), 156 | 30.0, (21.3-35.0), 21 | 30.0, (23.5-32.5), 17 | 30.0, (20.0-31.3), 28 | 0.403 |
| **Confidence about disease onset Median, (IQR), n** | | 69.5, (50.0-80.0), 156 | 52.5, (50.0-79.3), 21 | 60.0, (42.0-70.5), 17 | 64.5, (29.8-75.0), 28 | 0.329 |
| **Estimation of prevalence of CU in adult patients (%) Median, (IQR), n** | | 2.0, (1.0-4.0), 151 | 3.0, (2.0-4.8), 18 | 4.0, (2.0-5.0), 17 | 4.5, (1.8-6.3), 27 | 0.059 |
| **Percentage of CSU in CU adult patients Median, (IQR), n** | | 70.0, (60.0-80.0), 151 | 51.0, (13.0-75.8), 18 | 62.0, (33.5-70.5), 17 | 70.0, (47.5-77.0), 27 | 0.023  no significance after adjustment |
| **Percentage of concomitant CIndU in adults with CSU Median, (IQR), n** | | 30.0, (20.0-50.0), 151 | 21.0, (15.0-28.8), 18 | 39.0, (20.0-40.5), 17 | 30.0, (23.3-49.3), 27 | 0.031  G2 vs. G3 (0.033) |
| **Confidence about percentages for CSU and CIndU in adults Median, (IQR), n** | | 70.0, (50.0-80.0), 151 | 50.0, (31.0-70.0), 18 | 60.0, (50.0-72.5), 17 | 52.5, (32.8-75.0), 27 | 0.093 |
| **Estimation of prevalence of CU in pediatric patients (%) Median, (IQR), n** | | 1.0, (1.0-2.0), 115 | 2.0, (1.0-3.0), 15 | 2.0, (1.0-3.0), 13 | 2.5, (1.0-4.3), 23 | 0.052 |
| **Percentage of CSU in CU pediatric patients Median, (IQR), n** | | 60.0, (30.0-80.0), 115 | 20.5, (8.8-64.0), 15 | 62.0, (21.5-77.0), 13 | 56.5, (30.8-78.5), 23 | 0.255 |
| **Percentage of concomitant CIndU in pediatric patients with CSU Median, (IQR), n** | | 30.0, (11.8-50.0), 115 | 29.0, (10.3-47.5), 15 | 21.0, (13.5-40.0), 13 | 29.5, (14.3-42.5), 23 | 0.98**5** |
| **Confidence about percentages for CSU and CIndU in pediatric patients Median, (IQR), n** | | 60.0, (40.0-71.0), 115 | 50.0, (38.8-70.0), 15 | 50.0, (31.5-68.0), 13 | 50.0, (17.3-71.3), 23 | 0.561 |
| **Proportion of patients** **who** **have experienced severe impact on sleep, daily life, work, school or leisure activities Median, (IQR), n** | **CSU** | 50.0, (25.0-60.0), 155 | 50.0, (25.0-50.0), 21 | 35.0, (20.0-50.0), 14 | 40.0, (22.5-57.5), 26 | 0.595 |
|  | **CIndU** | 30.0, (20.0-40.0), 151 | 20.0, (10.0-30.0), 21 | 25.0, (10.0-40.0), 14 | 22.5, (10.0-38.8), 25 | 0.236 |
|  | **CSU+CIndU** | 50.0, (30.0-70.0), 151 | 60.0, (30.0-65.0), 20 | 45.0, (33.8-60.0), 14 | 50.0, (20.0-60.0), 25 | 0.243 |
| **Confidence about percentages for proportion of CU patients have experienced an impact Median, (IQR), n** | | 67.0, (50.0-74.8), 155 | 66.0, (50.0-80.0), 21 | 59.5, (54.8-76.3), 14 | 60.0, (31.5-77.0), 26 | 0.764 |

Abbreviations: IQR: interquartile range, n: subgroup within the total group, CSU: chronic spontaneous urticaria, CIndU: chronic inducible urticaria, CSU+CIndU: Patients affected by both CSU and CIndU, CU: chronic urticaria

**Suppl. Table S9. Comparisons based on personal CU history**

| **Personally affected by CU** | | **Yes (n=35)** | **No (n=191)** | **p-value** independent-Samples Mann-Whitney U Test |
| --- | --- | --- | --- | --- |
| **Disease onset CSU (in years) Median, (IQR), n** | | 30.0, (25.0-40.0), 35 | 30.0, (25.0-40.0), 191 | 0.428 |
| **Disease onset CIndU (in years) Median, (IQR), n** | | 20.0, (17.5-26.3), 35 | 25.0, (20.0-30.0), 191 | 0.082 |
| **Disease onset CSU+CIndU (in years) Median, (IQR), n** | | 30.0, (20.0-35.8), 35 | 30.0, (25.0-35.0), 191 | 0.333 |
| **Confidence about disease onset Median, (IQR), n** | | 62.5, (32.3-75.0), 35 | 68.0, (50.0-80.0), 191 | 0.239 |
| **Estimation of prevalence of CU in adult patients (%) Median (IQR), n** | | 3.0, (2.0-4.3), 34 | 2.0, (1.0-4.3), 182 | 0.333 |
| **Percentage of CSU in CU adult patients Median, (IQR), n** | | 70.0, (53.8-80.0), 34 | 70.0, (50.8-80.0), 182 | 0.915 |
| **Percentage of concomitant CIndU in adults with CSU Median, (IQR), n** | | 28.5, (20.0-60.0), 34 | 30.0, (20.0-45.3), 182 | 0.925 |
| **Confidence about percentages for CSU and CIndU in adults Median, (IQR), n** | | 51.0, (37.0-70.3), 34 | 70.0, (50.0-80.0), 182 | 0.036 |
| **Estimation of prevalence of CU in pediatric patients (%) Median, (IQR), n** | | 2.0, (1.0-3.3), 27 | 1.0, (1.0-2.0), 143 | 0.125 |
| **Percentage of CSU in CU pediatric patients Median, (IQR), n** | | 63.0, (30.0-80.0), 27 | 54.0, (25.8-75.5), 143 | 0.699 |
| **Percentage of concomitant CIndU in pediatric patients with CSU Median, (IQR), n** | | 40.0, (14.5-50.0), 27 | 27.5, (12.0-43.0), 143 | 0.292 |
| **Confidence about percentages for CSU and CIndU in pediatric patients Median, (IQR), n** | | 50.0, (13.8-61.5), 27 | 59.5, (40.0-71.0), 143 | 0.060 |
| **Proportion of patients** **who** **have experienced severe impact on sleep, daily life, work, school or leisure activities Median, (IQR), n** | **CSU** | 50.0, (30.0-60.0), 34 | 45.0, (25.0-60.0), 186 | 0.444 |
|  | **CIndU** | 30.0, (20.0-40.0), 33 | 30.0, (10.0-40.0), 181 | 0.371 |
|  | **CSU+CIndU** | 50.0, (35.0-70.0), 34 | 50.0, (30.0-61.3), 180 | 0.770 |
| **Confidence about percentages for proportion of CU patients have experienced an impact Median, (IQR), n** | | 60.0, (50.0-76.0), 34 | 66.5, (50.0-75.0), 186 | 0.772 |

Abbreviations: IQR: interquartile range, n: subgroup within the total group, CSU: chronic spontaneous urticaria, CIndU: chronic inducible urticaria, CSU+CIndU: Patients affected by both CSU and CIndU, CU: chronic urticaria

**Suppl. Table S10.** **Comparisons based on personal CU history and working at the UCARE**

| **Working at UCARE** | | **Working at UCARE and personally affected by CU (n=29)** | **Working at UCARE and not personally affected by CU (n=151)** | **p-value** independent-Samples Mann-Whitney U Test |
| --- | --- | --- | --- | --- |
| **Disease onset CSU (in years) Median, (IQR), n** | | 32.0, (28.0-37.5). 29 | 30.0, (25.0-40.0), 151 | 0.898 |
| **Disease onset CIndU (in years) Median, (IQR), n** | | 23.0, (18.0-30.0), 35 | 25.0, (20.0-30.0), 151 | 0.197 |
| **Disease onset CSU+CIndU (in years) Median, (IQR), n** | | 30.0, (22.5-36.5), 29 | 30.0, (25.0-35.0), 151 | 0.864 |
| **Confidence about disease onset Median, (IQR), n** | | 60.0, (33.0-75.0), 35 | 65.0, (50.0-79.0), 151 | 0.185 |
| **Estimation of prevalence of CU in adult patients (%) Median, (IQR), n** | | 3.0, (1.3-4.8), 28 | 2.0, (1.0-4.0), 144 | 0.384 |
| **Percentage of CSU in CU adult patients Median, (IQR), n** | | 70.0, (56.3-80.0), 28 | 70.0, (60.0-80.0), 144 | 0.843 |
| **Percentage of concomitant CIndU in adults with CSU Median, (IQR), n** | | 25.0, (20.0-55.0), 28 | 30.0, (20.0-40.0), 144 | 0.727 |
| **Confidence about percentages for CSU and CIndU in adults Median, (IQR), n** | | 56.0, (41.5-70.8), 28 | 67.0, (50.0-80.0), 144 | 0.119 |
| **Estimation of prevalence of CU in pediatric patients (%) Median, (IQR), n** | | 2.0, (1.0-3.0), 23 | 1.0, (1.0-2.0), 116 | 0.089 |
| **Percentage of CSU in CU pediatric patients Median, (IQR), n** | | 64.0, (30.0-80.0), 23 | 60.5, (26.8-80.0), 116 | 0.731 |
| **Percentage of concomitant CIndU in pediatric patients with CSU Median, (IQR), n** | | 40.0, (10.0-50.0), 23 | 25.0, (11.3-40.0), 116 | 0.390 |
| **Confidence about percentages for CSU and CIndU in pediatric patients Median, (IQR), n** | | 55.0, (20.0-71.0), 23 | 60.0, (40.5-72.8), 116 | 0.277 |
| **Proportion of patients** **who** **have experienced severe impact on sleep, daily life, work, school or leisure activities Median, (IQR), n** | **CSU** | 50.0, (20.0-60.0), 29 | 50.0, (27.5-60.0), 149 | 0.720 |
|  | **CIndU** | 27.5, (20.0-40.0), 28 | 30.0, (10.0-40.0), 145 | 0.415 |
|  | **CSU+CIndU** | 50.0, (35.0-70.0), 29 | 50.0, (30.0-70.0), 144 | 0.725 |
| **Confidence about percentages for proportion of CU patients have experienced an impact Median, (IQR), n** | | 70.0, (50.0-80.0), 29 | 69.0, (50.0-75.0), 149 | 0.975 |

Abbreviations: UCARE: Urticaria Centers of Reference and Excellence, IQR: interquartile range, n: subgroup within the total group, CSU: chronic spontaneous urticaria, CIndU: chronic inducible urticaria, CSU+CIndU: Patients affected by both CSU and CIndU, CU: chronic urticaria

**SUPPLEMENT FIGURES**

**Suppl. Figure S1. Questionnaire**

**1. About you**

The following questions gather some information about you.

**1.1.** **Please select your country**.* (drop-down list)

**1.2. What is your gender?***

- Male
- Female
- Diverse
- Other, please specify____
- Prefer not to answer

**1.3.** **What is your age? (years)_______**

**1.4.** **Which of the following describe your main medical specialty?***

- Allergy/Allergology or Allergy/Clinical Immunology
- General Practice/Primary Care
- Internal Medicine
- Dermatology
- Pediatrics
- I am not a physician
- Other, please specify_____

**1.5.** **For how many years have you been in clinical practice?***

- ____years
- I am not in clinical practice

**1.6.** **What is your primary place of work?*** (at this place you spend more than 50% of your working time)

ο University/Training hospital
ο Public hospital 
ο Private hospital
ο Private practice (office)
ο Other, please specify……

**1.6.1 is your primary place of work connected to a Urticaria Center of Reference and Excellence (UCARE)?***

ο Yes
ο No

**2. Characteristics of your patients**

**2.1.** **Are you a physician who sees patients with chronic urticaria (CU) in your clinical practice?***

- Yes, I see only adult patients (≥18 years) with CU
- Yes, I see only pediatric patients (<18 years) with CU
- Yes, I see both adult (≥18 years) and pediatric patients (<18 years) with CU
- No, I do not see patients with CU

**3. Estimating the prevalence of CU**

Chronic urticaria is a heterogeneous disease. It is classified as spontaneous (CSU, when definite and specific external trigger is absent) or inducible (CIndU, when definite and specific external trigger is present, e.g. cold).

| Classification of urticaria | | |
| --- | --- | --- |
| Types of urticaria | Characteristics | Subtypes |
| Acute urticaria | Duration ≤6 weeks |  |
| Chronic spontanous urticaria (CSU) | Spontaneous appearance of wheals, angioedema, or both for >6 weeks, (no definite eliciting factor involved) | - Known cause (e.g. autoimmune disease) - Unknown cause |
| Chronic inducible urticaria (CIndU) | Symptomes for >6 weeks, specific definite factor involved | - Symptomatic dermographism - Cold urticaria - Delayed pressure urticaria - Solar urticaria - Heat urticaria - Vibratory angioedema - Cholinergic urticaria - Contact urticaria - Aquagenic urticaria |

Source: Zuberbier T,  Altrichter S,  Bauer S, et al.  S3 Guideline Urticaria. Part 1: Classification and diagnosis of urticaria – German-language adaptation of the international S3 Guideline. *JDDG: Journal der Deutschen Dermatologischen Gesellschaft*.  2023; 21: 81–93.

Please note that the following questions now ask you about your estimates related to the prevalence of CU in your country. This may be a difficult exercise, but your best estimate is fine.

**3.1. What is your estimation of the average age of disease onset?*** (Please try to provide an average estimate considering all patients with CU in your country, and not only the patients you usually see in your clinical practice.)

CSU: years____

CindU: years_____

Both CSU and CindU in the same patient: years_____

**3.2. How confident are you in the estimates provided about the average age of disease onset?*** If you have a high confidence, choose a high number. If you have a low confidence, choose a low number.


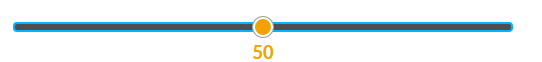


**3.3. What is your estimate of the prevalence (%) of CU in adults in your country?*** Please enter a number between **0 and 10.** For example, if you enter 3, it means that you estimate that 3% of adults in your country have CU. ___________

If you think the prevalence for CU in your country is higher than 10% please put your estimation here____

**3.4. Among adult patients with CU in your country, what percentage do you estimate have CSU**?* Please enter a number between **0 and 100.** For example, if you enter 40, it means that you estimate that 40% of adult CU patients have CSU (with or without concomitant CIndU) and 60% have isolated CIndU. ___________

**3.5. Among adult patients with CSU in your country, what percentage do you estimate have concomitant CIndU?*** Please enter a number between **0 and 100.** For example, if you enter 40, it means that you estimate that 40% of adult CSU patients have concomitant CIndU and 60% do not have concomitant CIndU._________

**3.6. How confident are you about your estimated percentages for CSU and CindU?*** If you have a high confidence, choose a high number. If you have a low confidence, choose a low number.


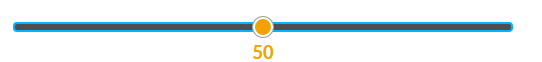


**3.7. What is your estimate of the prevalence (%) of CU in pediatric patients (<18 years) in your country?*** Please enter a number between **0 and 10.** For example, if you enter 3, it means that you estimate that 3% of children in your country have CU. ___________

If you think the prevalence for CU in your country is higher than 10% please put your estimation here____

**3.8. Among pediatric patients (<18 years) with CU in your country, what percentage do you estimate have CSU**?* Please enter a number between 0 and 100. For example, if you enter 40, it means that you estimate that 40% of pediatric CU patients have CSU (with or without concomitant CIndU) and 60% have isolated CIndU. ___________

**3.9. Among pediatric patients (<18 years) with CSU in your country, what percentage do you estimate have concomitant CIndU**?* Please enter a number between 0 and 100. For example, if you enter 40, it means that you estimate that 40% of pediatric CSU patients have concomitant CIndU and 60% do not have concomitant CIndU.________

**3.10. How confident are you about your estimated percentages  for CSU and CindU among pediatric patients (< 18 years)?*** If you have a high confidence, choose a high number. If you have a low confidence, choose a low number.


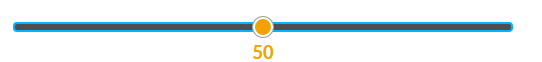


**4. Estimating the burden of CU**

Definition of burden of disease: The term burden of disease generally describes the total, cumulative consequences of a defined disease or a range of harmful diseases with respect to disabilities in a community. These consequences include health, social aspects, and costs to society. The gap between an ideal situation, where everyone lives free of disease and disability, and the cumulated current health status, is defined as the burden of disease.

Hessel, F. (2008). Burden of Disease . In: Kirch, W. (eds) Encyclopedia of Public Health. Springer, Dordrecht. <https://doi.org/10.1007/978-1-4020-5614-7_297>

**4.1. What are the most affected aspects of patient’s life due to CSU?** (please order, 1-highly affected, 8-least affected)

- Physical activities
- Sleep
- Spare time
- Social relationships
- Eating behavior
- Mental status
- Appearance (e.g. choice of clothing, cosmetic, avoidance of public)
- Work and/or school

**4.2. What are the most affected aspects of patient’s life due to CIndU?** (please order, 1-highly affected, 8-least affected)

- Physical activities
- Sleep
- Spare time
- Social relationships
- Eating behavior
- Mental status
- Appearance (e.g. choice of clothing, cosmetic, avoidance of public)
- Work and/or school

**4.3. What are the most affected aspects of patient’s life due to CSU and CindU in the same patient?** (please order, 1-highly affected, 8-least affected)

- Physical activities
- Sleep
- Spare time
- Social relationships
- Eating behavior
- Mental status
- Appearance (e.g. choice of clothing, cosmetic, avoidance of public)
- Work and/or school

**4.4. What proportion of CU patients have experienced an impact on sleep, daily life, work, school or leisure activities?*** Please enter a number between 0 and 100 (the sum of your answers should be 100%). Please try to provide an average estimate considering all patients with CU in your country, and not only the patients you usually see in your clinical practice. **This may be a difficult exercise, but your best estimate is fine.**

**CSU alone**

- Severe impact_________
- Moderate impact_______
- Mild/No impact______

**CindU alone**

- Severe impact_________
- Moderate impact_______
- Mild/No impact______

**Both CSU and CindU in the same patient**

- Severe impact_________
- Moderate impact_______
- Mild/No impact______

**4.5.** **How confident are you in the estimated impact on sleep, daily life, work, school or leisure activities?***

If you have a high confidence, choose a high number. If you have a low confidence, choose a low number.


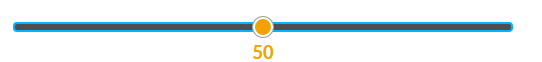


**6. Now we would like to ask you whether you yourself have or have ever had a form of urticaria and how you yourself assess and experience your condition. If you have never had urticaria, the questionnaire ends here and we thank you for your participation.**

**Assessment of your experience as an urticaria patient**

The following questions now aim to assess the most bothersome CU symptoms for you as a patient.

**6.1. Do you have or have you previously had acute urticaria (≤6 weeks of duration)?***

- Yes
- No

**6.2. Do you have or have you previously had CU (>6 weeks of duration)?***

- Yes
- No

**6.2.1.** **Considering the international urticaria classification, which of the following options best describes your CU**?*

- Chronic spontaneous urticaria (CU)
- Chronic inducible urticaria (CindU)
- Both
- I don’t know/I am not sure

| Classification of urticaria | | |
| --- | --- | --- |
| Types of urticaria | Characteristics | Subtypes |
| Acute urticaria | Duration ≤6 weeks |  |
| Chronic spontanous urticaria (CSU) | Spontaneous appearance of wheals, angioedema, or both for >6 weeks, (no definite eliciting factor involved) | - Known cause (e.g. autoimmune disease) - Unknown cause |
| Chronic inducible urticaria (CIndU) | Symptomes for >6 weeks, specific definite factor involved | - Symptomatic dermographism - Cold urticaria - Delayed pressure urticaria - Solar urticaria - Heat urticaria - Vibratory angioedema - Cholinergic urticaria - Contact urticaria - Aquagenic urticaria |

Source: Zuberbier T,  Altrichter S,  Bauer S, et al.  S3 Guideline Urticaria. Part 1: Classification and diagnosis of urticaria – German-language adaptation of the international S3 Guideline. *JDDG: Journal der Deutschen Dermatologischen Gesellschaft*.  2023; 21: 81–93.

**6.2.2 for how many years have you had CU?*** Years___

**6.2.3 how would you rate the activity of your disease?***

- High activity_________
- Moderate activity_______
- Low activity_____

**6.2.4.** **What are the most frequent CU symptoms you experience(d)?*** Please choose all that apply

- Itch
- Wheals
- Angioedema
- Other symptoms, please specify

**6.2.5.** **What are the CU symptom(s) that, when present, bother(ed) you the most?***

- Itch
- Wheals
- Angioedema
- Other symptoms, please specify

**7. In the event that questions arise concerning your answers to this survey, we would like to be able to contact you.**

**I agree that I may be contacted again at a later date.**

**□ yes □ no**

**7.1 for the purpose of participating in further questionnaire studies or recommending colleagues:**

**□ yes □ no**

**7.2 If yes: Please provide your e-mail address here___________________________**

**7.3 What is your name?**

**It is important to us to also include the views of GPs in this project. We would therefore like to ask you to forward the link to this survey to all GPs you know. Thank you for your support!**

**8. If you have any comments or observations before submitting the survey, please provide them here.______**

**Suppl. Figure S2** **Flowchart participants**

Total number of participants who were qualified n=234

Participants who completed the questionnaire n=244

Participants who started the questionnaire n=522

Participants who disqualified while filling out the questionnaire due to:

- Did not agree to participate n=6 and /or
- weren’t physicians n=4 and/or
- didn’t treat patients with CU n=7

Participants who didn’t complete the questionnaire n=278
